# Frequency and risk factors for failed weaning from supplemental oxygen therapy after general anesthesia at a postanesthesia care unit: a retrospective cohort study

**DOI:** 10.1186/s12871-023-02192-z

**Published:** 2023-07-07

**Authors:** Yu Jeong Bang, I Hyun Park, Heejoon Jeong

**Affiliations:** 1 Department of Anesthesiology and Pain Medicine, Samsung Medical Center, Sungkyunkwan University School of Medicine, Seoul, South Korea; 2 81 Irwon-ro, Gangnam-gu, Seoul, 06351 South Korea

**Keywords:** Supplemental oxygen, Weaning, Risk factor, Postanesthesia care unit

## Abstract

**Background:**

Patients are administered supplemental oxygen upon emergence from general anesthesia against the risk of hypoxia. However, few studies have assessed the weaning from supplemental oxygen therapy. This study investigated the frequency and risk factors of failure to discontinue supplemental oxygen at a postanesthesia care unit (PACU).

**Methods:**

This retrospective cohort study was conducted in a tertiary hospital. We reviewed the medical records of adult patients admitted to the PACU after general anesthesia for elective surgery between January 2022 and November 2022. The primary endpoint was the frequency of failed weaning from supplemental oxygen therapy at PACU. A failed weaning was defined as oxygen saturation (SpO_2_) < 92% after discontinuing oxygen administration. The rate of failed discontinuation of supplemental oxygen at the PACU was assessed. Demographics, intraoperative, and postoperative factors were explored to determine potential associations with failed weaning from supplemental oxygen therapy using logistic regression analysis.

**Results:**

We analyzed 12,109 patients. We identified 842 cases of failed weaning from supplemental oxygen therapy, with a frequency of 1:14 (95% confidence interval [CI], 1:15–1:13). Risk factors that showed the strongest associations with failed weaning included postoperative hypothermia (odds ratio [OR], 5.42; 95% CI, 4.40–6.68; *P* < 0.001), major abdominal surgery (OR, 4.04; 95% CI, 3.29–4.99; *P* < 0.001), and preoperative SpO_2_ < 92% in room air (OR, 3.15; 95% CI, 2.09–4.64; *P* < 0.001).

**Conclusion:**

In the analysis of more than 12,000 general anesthetics, an overall risk of failed weaning from supplemental oxygen therapy of 1:14 was observed. The identified risk factors may help determine the discontinuation of supplemental oxygen administration at PACU.

**Trial registration:**

Not applicable.

## Background

Most patients are administered supplemental oxygen during recovery from general anesthesia in a postanesthesia care unit (PACU). Although several papers suggested that routine administration of supplemental oxygen to all patients after general anesthesia is unnecessary, the recent practice guideline for postanesthetic care reported by the American Society of Anesthesiologists Task Force recommends the administration of supplemental oxygen during emergence and recovery for patient safety [1–3].

Patients recovering from general anesthesia are prone to hypoxemia owing to the residual effects of anesthetics, opioids, and neuromuscular blockers [4]. Although the widespread use of pulse oximetry and neuromuscular monitoring has contributed to the reduction of hypoxemia at the PACU [5], empirical oxygen supplementation is still provided to many patients during recovery from general anesthesia. Most successfully discontinue supplemental oxygen therapy; however, several patients fail to wean from supplemental oxygen therapy and eventually discharge with oxygen supplementation. However, in most hospitals, vigilant oxygenation monitoring is unavailable in the general ward. Therefore, the inappropriate discontinuation of supplemental oxygen or unexpected failure of oxygenation after cessation of oxygen administration may be detrimental to patient safety.

Successful weaning from oxygen therapy in the PACU is determined by various factors, including comorbidities, surgical characteristics, anesthesia technique, or postoperative management. To our knowledge, no studies have investigated the incidence and risk factors for failed weaning from oxygen therapy in the PACU. Most previous studies dated back to the 1990s, when anesthesia and PACU care differed from today [6, 7], or focused on postoperative hypoxemia regardless of supplemental oxygen therapy [8–10].

We aimed to estimate the frequency of failed weaning from supplemental oxygen therapy in the PACU in patients who underwent general anesthesia, leveraging a large number of cases. Moreover, we elucidated the significant risk factors for failed weaning from supplemental oxygen therapy to help make a precise decision during the discontinuation of supplemental oxygen at the PACU.

## Methods

### Study design and ethical statements

This study was a retrospective cohort analysis of patients admitted to the PACU after general anesthesia at a single tertiary medical center in South Korea. The study was approved by the Institutional Review Board of the Samsung Medical Center (approval No: SMC 2022-12-107; date of approval: December 23, 2022), which waived the need for written informed consent from participants due to the non-interventional study design. We complied with the Strengthening the Reporting of Observational Studies in Epidemiology checklist for reporting this study [11]. All methods were performed in accordance with the ethical principles of the 1964 Declaration of Helsinki and its later amendments and were carried out following the approved guidelines.

### Study population

We collected the electronic medical records of patients aged > 19 years who were admitted to the PACU after elective surgery under general anesthesia between January 1, 2022, and November 30, 2022. A total of 24,823 adult patients were assessed for eligibility. Before accessing the data, the following exclusion criteria were used: (1) thoracic surgery requiring postoperative chest tube drainage; (2) airway surgery; (3) general anesthesia without endotracheal intubation, that is, mask-holding or supraglottic airway devices; (4) general anesthesia for procedures including esophagogastroscopy, colonoscopy, and bronchoscopy; (5) duration of surgery < 1 h ; (6) PACU stay < 30 min; and (7) no supplemental oxygen therapy at the PACU.

### Data collection

We obtained the demographic characteristics, intraoperative and anesthetic data, and PACU data from the electronic medical record and clinical data warehouse of the Samsung Medical Centre. Baseline characteristics included age, sex, body mass index, smoking within six months from the operation day, American Society of Anesthesiologists (ASA) physical status, preoperative peripheral oxygen saturation (SpO_2_) in room air, and comorbidities including hypertension, diabetes mellitus, chronic obstructive respiratory disease (COPD), cardiovascular disease, and cerebrovascular disease. Cardiovascular disease included coronary artery disease and arrhythmia, while cerebrovascular disease included a history of cerebral infarction and cerebral hemorrhage.

Intraoperative and anesthesia data included the type of anesthesia (inhaled or intravenous anesthesia), type of surgery (major abdominal surgery or else), duration of surgery, volume of fluid administered, transfusion, estimated blood loss, and administration of sugammadex during emergence. Major abdominal surgery included exploratory laparotomy, gastrointestinal surgery, hepato-pancreato-biliary surgery, nephrectomy, adrenalectomy, prostatectomy, hysterectomy, and gynecologic debulking surgery.

PACU data included the flow of oxygen applied to the patients, SpO_2_, pain intensity score measured using a numeric rating scale (NRS; 0 = no pain, 10 = worst pain imaginable), postoperative nausea and vomiting, and core temperature measured at the tympanic membrane.

### Protocol for weaning from supplemental oxygen and definition of failed weaning

During emergence from general anesthesia, neuromuscular blockade was reversed with pyridostigmine when the train-of-four count were 3 or greater, or sugammadex when the train-of-four counts were 2 or less. The attending anesthesiologist performed extubation after confirming the patient restored consciousness, muscle power, and spontaneous breathing. After successful extubation, patient was transferred to the PACU without oxygen administration.

**Fig. 1:**
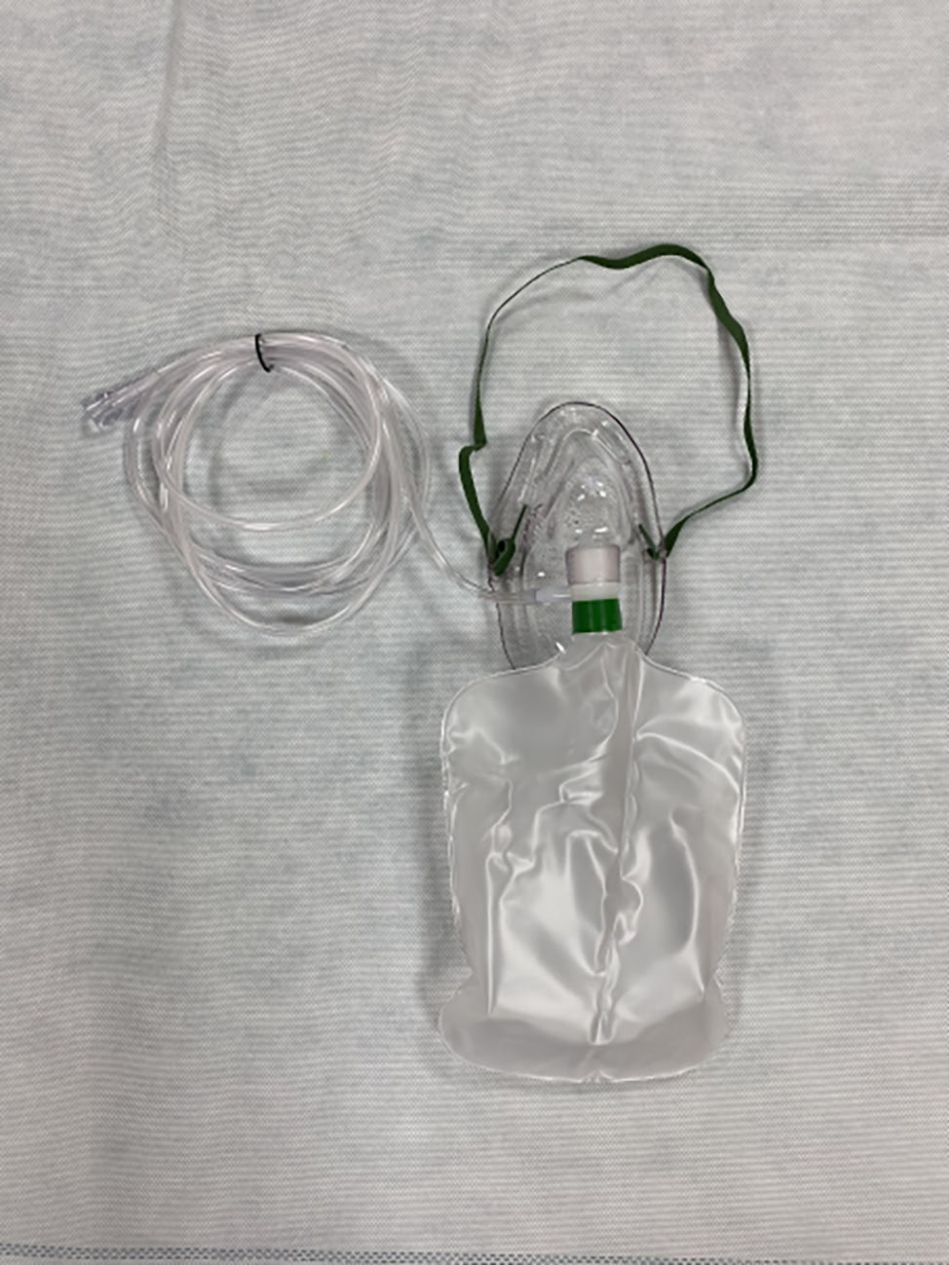
Non-rebreather oxygen mask

Upon arrival at the PACU, the patient received supplemental oxygen at 10 L·min^− 1^ through a non-rebreather mask (Hudson RCI® High-Concentration Oxygen Mask, Teleflex, Morrisville, NC, USA) (Fig. 1). If the SpO_2_was maintained at > 98% for 10 min without any evidence of oxygenation impairment, we decreased the flow of supplemental oxygen to 5 L/min and maintained it for 10 min. If a patient showed sufficient oxygenation (SpO_2_ > 98%) and stable hemodynamics, supplemental oxygen was discontinued. If the SpO_2_ decreased below 92% in room air after the discontinuation of supplemental oxygen, the attending nurse encouraged the patient to perform forced deep breathing. If the SpO_2_ was not maintained at over 92% despite more than three attempts to encourage forced breathing, the anesthesiologist in charge of the PACU resumed oxygen administration at a flow of ≥ 5 L/min through a non-rebreather mask. Patients who failed to wean from supplemental oxygen therapy were transferred to the general ward or the intensive care unit with oxygen supplementation and SpO_2_ monitoring.

We defined failed weaning from supplemental oxygen therapy as when SpO_2_ was not maintained over 92% in room air after the discontinuation of supplemental oxygen administration.

### Study outcome and measurements

The primary endpoint was the frequency of failed weaning from supplemental oxygen therapy in the PACU in adult patients who underwent general anesthesia. Potential cases of failed weaning from supplemental oxygen were identified through an electronic search of PACU medical records. All potential electronically identified cases were reviewed by two independent investigators (Y. J. B. and I. H. P.). All discrepancies were resolved by consensus between the two investigators. We calculated the frequency of failed weaning from supplemental oxygen and its 95% confidence interval (CI) for adult patients who underwent general anesthesia.

### Potential risk factors

We assessed the following potential risk factors for failed weaning from supplemental oxygen therapy after general anesthesia based on the plausibility and risk factors that were reported in the previous literature [12–16]: age > 65 years, male sex, body mass index ≥ 30 kg/m^2^, hypertension, diabetes mellitus, COPD, ASA physical status ≥ III, smoking history within six months from the operation day, preoperative SpO_2_ < 92% in room air, major abdominal surgery, duration of operation over two hours, intraoperative fluid administration over 1,500 mL, intraoperative transfusion, estimated blood loss over 400 mL, inhaled anesthesia, use of sugammadex during emergence, postoperative nausea and vomiting, moderate to severe postoperative pain of NRS ≥ 4/10, and hypothermia at the PACU (core temperature measured at the tympanic membrane < 36.0 ℃).

### Statistical analysis

Continuous variables are summarized as medians with interquartile ranges. Categorical variables are summarized as frequency (%). The incidence is presented as point estimates with a 95% Wilson score CI. Logistic regression analysis using 19 potential risk factors was performed to estimate the odds ratios (ORs) with corresponding 95% CIs for failed weaning from supplemental oxygen therapy at the PACU. Before logistic regression, we evaluated multicollinearity among potential risk factors using the variance inflation factors. The variables that were associated (*P* < 0.2) during the univariate logistic regression were entered into a multivariate stepwise (backward elimination) logistic regression. A logistic regression model was established to identify risk factors associated with weaning failure. All statistical tests were 2-sided, and *P* < 0.05 was considered significant. Statistical analyses were performed using R software (version 4.3.1; R Foundation for Statistical Computing, Austria).

Before the analysis, we expected to identify approximately 13,000 patients who were admitted to the PACU after general anesthesia at our institute. The frequency of failed weaning from supplemental oxygen therapy in the pilot study was observed as 5.5%. Assuming a 5% incidence of failed weaning from supplemental oxygen therapy, we determined that analysis of 13,000 cases of general anesthesia would allow for a 99% probability of obtaining a 95% Wilson CI half-width for the incidence of 0.2%.

## Results

**Fig. 2:**
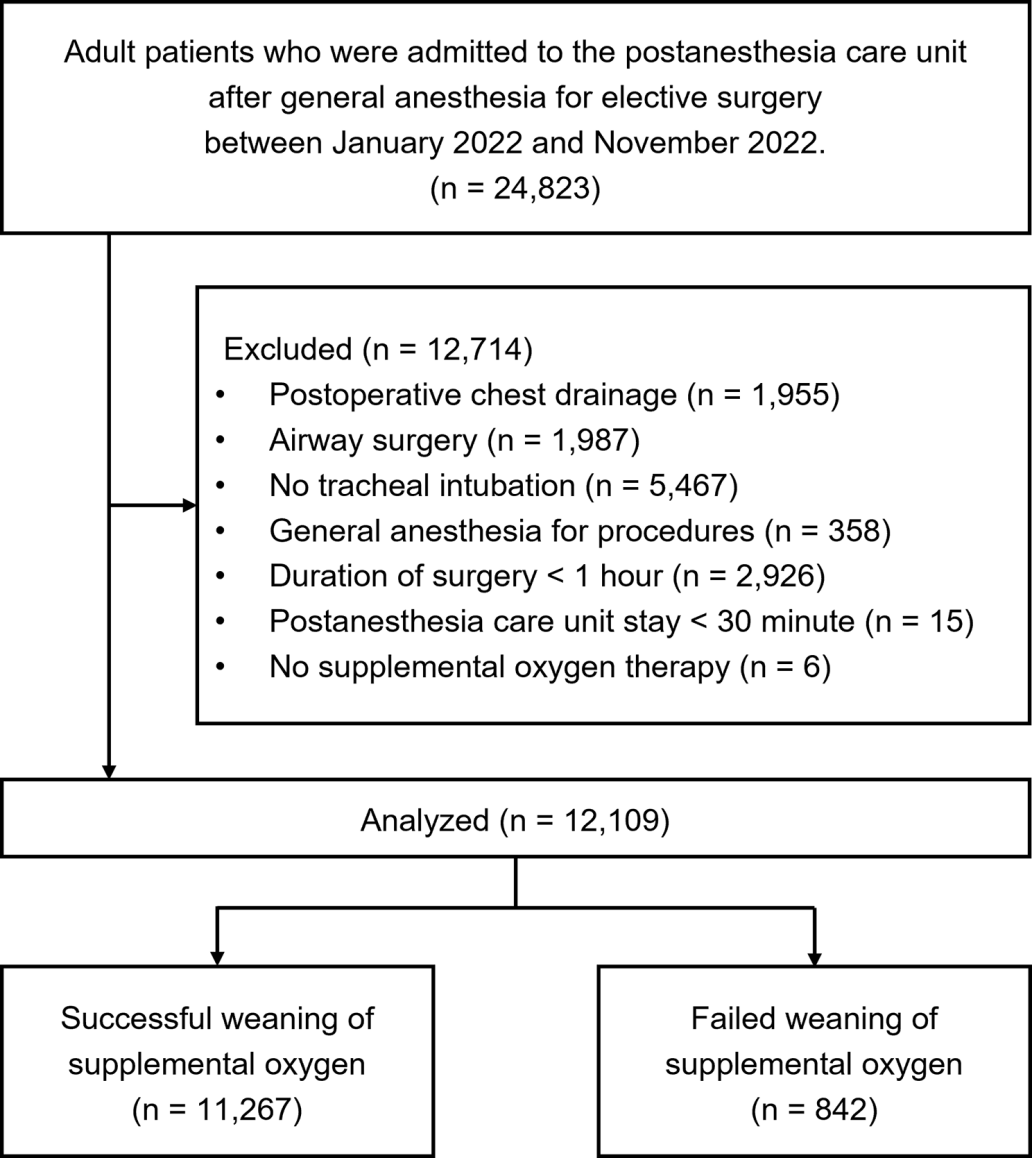
Flow diagram of the study

We identified 24,823 adult patients admitted to the PACU after general anesthesia between January 1, 2022, and November 30, 2022. Of these, 12,714 cases were excluded according to the exclusion criteria determined before analysis as follows (Fig. 2): (1) thoracic surgery requiring postoperative chest tube drainage (n = 1,955), (2) airway surgery (n = 1,987), (3) general anesthesia without endotracheal intubation (n = 5,467), (4) general anesthesia for the procedure (n = 358), (5) duration of surgery < 1 h (n = 2,926), (6) PACU stay < 30 min (n = 15), and (7) no supplemental oxygen therapy at the PACU (n = 6).

Upon review of the medical records, there were 842 cases of failed weaning from supplemental oxygen therapy; the incidence of failed weaning from supplemental oxygen therapy was 1:14 (95% CI, 1:15–1:13). The characteristics of all patients, as well as the subset who failed weaning from supplemental oxygen therapy at the PACU, are presented in Table 1.

**Table 1:** Patient characteristics of patients

Variable	Overall (n = 12,109)	Groups		
		Failed weaning (n = 842)	Successful weaning (n = 11,267)	SMD
Age, years	57 (45–66)	64 (53–72)	56 (45–66)	0.441
Sex, male	5,150 (43)	498 (59)	4,652 (41)	0.363
Body mass index, kg/m^2^	24.1 (21.8–26.5)	24.8 (22.7–27.3)	24.0 (21.8–26.4)	0.243
Comorbidities				
Hypertension	3,266 (27)	350 (42)	2,916 (26)	0.336
Diabetes mellitus	1,669 (14)	209 (25)	1,460 (13)	0.307
Chronic obstructive pulmonary disease	290 (2)	43 (5)	247 (2)	0.156
ASA physical status ≥ III	1,185 (10)	170 (20)	1,015 (9)	0.321
Smoking history within 6 months	1,342 (11)	142 (17)	1,200 (11)	0.181
Preoperative SpO_2_ < 92% in room air	189 (2)	41 (5)	148 (1)	0.206
Type of surgery, major abdominal surgery*	5,756 (48)	629 (75)	5,127 (46)	0.625
Duration of surgery, min	124 (92–167)	155 (114–203)	122 (91–164)	0.376
Intraoperative fluid infusion, mL	750 (550–1100)	1100 (800–1450)	750 (550–1000)	0.477
Intraoperative transfusion	246 (2)	32 (4)	214 (2)	0.114
Estimated blood loss, mL	50 (50–150)	150 (80–250)	50 (50–150)	0.421
Type of anesthesia				0.214
Inhaled anesthesia	8,956 (74)	692 (82)	8,264 (73)	
Total intravenous anesthesia	3,153 (26)	150 (18)	3,003 (27)	
NRS pain score at the PACU	4 (3–5)	5 (4–5)	4 (3–5)	0.347
Postoperative nausea and vomiting	849 (7)	60 (7)	789 (7)	0.005
Core temperature at the PACU, ℃	36.3 (36.0–36.3)	36.1 (35.9–36.3)	36.3 (36.1–36.4)	0.488

Data presented as median (interquartile range) or frequency (%). *Major abdominal surgery included exploratory laparotomy, gastrointestinal surgery, hepato-pancreato-biliary surgery, nephrectomy, adrenalectomy, prostatectomy, hysterectomy, and gynecologic debulking surgery

SMD = standard mean difference; ASA = American Society of Anesthesiologists; SpO_2_ = oxygen saturation measured by pulse oximetry; NRS = numeric rating scale; PACU = postanesthesia care unit

We investigated 19 different characteristics for potential association with failed weaning from supplemental oxygen therapy in the PACU. Univariate analyses demonstrated age > 65 years (OR, 2.20; 95% CI, 1.91–2.53; *P* < 0.001), male sex (OR, 2.06; 95% CI, 1.79–2.38; *P* < 0.001), body mass index ≥ 30 kg/m^2^ (OR, 1.44; 95% CI, 1.12–1.82; *P* = 0.003), hypertension (OR, 2.04; 95% CI, 1.76–2.35; *P* < 0.001), diabetes mellitus (OR, 2.22; 95% CI, 1.88–2.61; *P* < 0.001), COPD (OR, 2.40; 95% CI, 1.70–3.31; *P* < 0.001). ASA physical status ≥ III (OR, 2.56 95% CI, 2.13–3.05; *P* < 0.001), Smoking history within six months (OR, 1.70; 95% CI, 1.40–2.05; *P* < 0.001), preoperative SpO2 < 92% in room air (OR, 3.85; 95% CI, 2.67–5.42; *P* < 0.001), major abdominal surgery (OR, 3.54; 95% CI, 3.02–4.16; *P* < 0.001), duration of operation over two hours (OR, 2.31; 95% CI, 1.98–2.70; *P* < 0.001), intraoperative fluid infusion over 1,500 mL (OR, 2.65; 95% CI, 2.21–3.16; *P* < 0.001), intraoperative transfusion (OR, 2.04; 95% CI, 1.37–2.93; *P* < 0.001), estimated blood loss over 400 mL (OR, 2.51; 95% CI, 1.94–3.22; *P* < 0.001), inhaled anesthesia (OR, 1.68; 95% CI, 1.40–2.02; *P* < 0.001), moderate to severe postoperative pain of NRS ≥ 4/10 (OR, 1.98; 95% CI, 1.66–2.38; *P* < 0.001), and hypothermia during the PACU stay (OR, 2.49; 95% CI, 2.14–2.88; *P* < 0.001) as potential risk factors for failed weaning from supplemental oxygen therapy (Table 2).

**Table 2:** Risk factors for failed weaning from supplemental oxygen on univariate analysis

Variable	Odds ratio (95% CI)	*P*-value
Age > 65 years	2.20 (1.91 to 2.53)	< 0.001
Male sex	2.06 (1.79 to 2.38)	< 0.001
Body mass index ≥ 30 kg/m^2^	1.44 (1.12 to 1.82)	0.003
Hypertension	2.04 (1.76 to 2.35)	< 0.001
Diabetes mellitus	2.22 (1.88 to 2.61)	< 0.001
Chronic obstructive pulmonary disease	2.40 (1.70 to 3.31)	< 0.001
ASA physical status ≥ III	2.56 (2.13 to 3.05)	< 0.001
Smoking history within 6 months	1.70 (1.40 to 2.05)	< 0.001
Preoperative SpO_2_ < 92% in room air	3.85 (2.67 to 5.42)	< 0.001
Major abdominal surgery*	3.54 (3.02 to 4.16)	< 0.001
Duration of surgery > 2 h	2.31 (1.98 to 2.70)	< 0.001
Intraoperative fluid infusion > 1,500 mL	2.65 (2.21 to 3.16)	< 0.001
Intraoperative transfusion	2.04 (1.37 to 2.93)	< 0.001
Estimated blood loss > 400 mL	2.51 (1.94 to 3.22)	< 0.001
Inhaled anesthesia	1.68 (1.40 to 2.02)	< 0.001
Use of sugammadex during the emergence	0.94 (0.81 to 1.10)	0.455
Postoperative pain of NRS ≥ 4/10	1.98 (1.66 to 2.38)	< 0.001
Postoperative nausea and vomiting	1.02 (0.77 to 1.33)	0.893
Hypothermia during the PACU stay†	2.49 (2.14 to 2.88)	< 0.001

*Major abdominal surgery included exploratory laparotomy, gastrointestinal surgery, hepato-pancreato-biliary surgery, nephrectomy, adrenalectomy, prostatectomy, hysterectomy, and gynecologic debulking surgery. **†**Core temperature measured at the tympanic membrane below 36.0 ℃

CI = confidence interval; ASA = American Society of Anesthesiologists; SpO_2_ = oxygen saturation measured by pulse oximetry; NRS = numeric rating scale; PACU = postanesthesia care unit

**Fig. 3:**
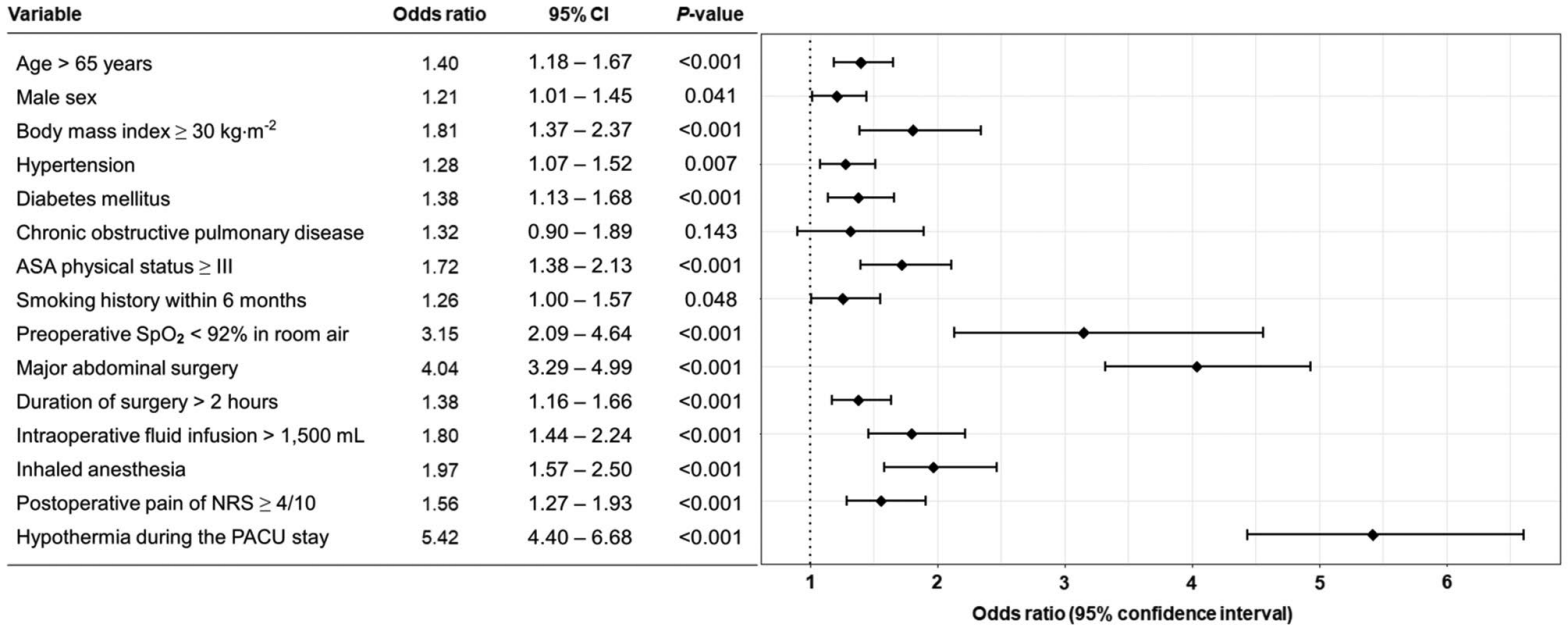
Risk factors for failed weaning from supplemental oxygen therapy

On multivariate logistic regression analysis, variables with significant association with failed weaning from supplemental oxygen therapy were age > 65 years (OR, 1.40; 95% CI, 1.18–1.67; *P* < 0.001), male sex (OR, 1.21; 95% CI, 1.01–1.45; *P* = 0.041), body mass index ≥ 30 kg/m^2^ (OR, 1.81; 95% CI, 1.37–2.37; *P* < 0.001), hypertension (OR, 1.28; 95% CI, 1.07–1.52; *P* = 0.007), diabetes mellitus (OR, 1.38; 95% CI, 1.13–1.68; *P* < 0.001), ASA physical status ≥ III (OR, 1.72; 95% CI, 1.38–2.13; *P* < 0.001), smoking history within six months (OR, 1.26; 95% CI, 1.00–1.57; *P* = 0.048), preoperative SpO_2_ < 92% in room air (OR, 3.15; 95% CI, 2.09–4.64; *P* < 0.001), major abdominal surgery (OR, 4.04; 95% CI, 3.29–4.99; *P* < 0.001), duration of operation over 2 h (OR, 1.38; 95% CI, 1.16–1.66; *P* < 0.001), intraoperative fluid administration over 1,500 ml (OR, 1.80; 95% CI, 1.44–2.24; *P* < 0.001), inhaled anesthesia (OR, 1.97, 95% CI, 1.57–2.50; *P* < 0.001), moderate to severe postoperative pain of NRS ≥ 4/10 (OR, 1.56; 95% CI, 1.27–1.93; *P* < 0.001), and hypothermia during the PACU stay (OR, 5.42; 95% CI, 4.40–6.68; *P* < 0.001) (Fig. 3).

## Discussion

In this study including more than 12,000 cases of general anesthesia, we examined the frequency of failed weaning from supplemental oxygen therapy, which was 6.95%. We also identified risk factors for failed weaning from supplemental oxygen therapy; postoperative hypothermia, major abdominal surgery, and preoperative SpO_2_ < 92% in room air showed the strongest association with failed weaning from supplemental oxygen therapy in the PACU.

The anesthesiologists in charge of the PACU often decide to discontinue supplemental oxygen based on their own experience due to the paucity of literature on this subject. There are several studies on hypoxemia in the PACU, but they are old to apply to recent medical practice [5–7]. Therefore, we need updated evidence reflecting recent advances in medical therapy and monitoring skills. To our knowledge, this is the first investigation to focus on the incidence and predictors of failed weaning from supplemental oxygen at the PACU in patients who underwent general anesthesia. This study has important clinical implications that alert the need for precise decisions during the weaning from supplemental oxygenation therapy in patients with more risk factors. Additionally, correcting modifiable risk factors can reduce the incidence of failed weaning.

The literature on postoperative hypoxemia reported that the incidence of desaturation at the PACU as 12 – 30% depending upon the cut-off of 94–92% SpO_2_ [17]. Meanwhile, we observed a 6.95% incidence of desaturation in room air after the discontinuation of supplemental oxygen. This discrepancy is due to the different time points of hypoxemia measurement between studies. As the risk of postoperative hypoxemia gradually decreases as the patient recovers from anesthesia, the patients are more prone to hypoxemia during the initial phase of recovery [9, 10]. While published studies have assessed postoperative hypoxemia immediately after arrival at the PACU, we investigated hypoxemia after the discontinuation of oxygen administration. Supplemental oxygen therapy in the early period of recovery, when the risk of postoperative hypoxemia was relatively high, could contribute to a lower incidence of postoperative hypoxemia in this study. This is consistent with previous studies that reported that oxygen supplementation immediately after general anesthesia could reduce the risk of postoperative hypoxemia during the PACU stay [9, 10].

The major risk factors that showed an OR greater than two in this analysis included postoperative hypothermia, major abdominal surgery, and preoperative SpO_2_ < 92% in room air. Postoperative hypothermia causes various postoperative complications, such as cardiac ischemia, coagulopathy, or wound infection [18], and to prolong recovery by augmenting the anesthetic effects, delaying drug elimination, or impairing cognitive function [19]. In addition, postoperative hypothermia triggers shivering which increases oxygen consumption [20] and shifts the oxygen-hemoglobin dissociation curve to left, which may lead to peripheral tissue hypoxia and desaturation. The adverse effects of postoperative hypothermia can impair the recovery of major functions including respiration and oxygenation, resulting in failed weaning from supplemental oxygen therapy at the PACU.

Major abdominal surgery can induce intraoperative alveolar collapse and postoperative diaphragmatic dysfunction, thus causing postoperative pulmonary complications, especially atelectasis, leading to postoperative hypoxemia [21]. We also identified that major abdominal surgery is a significant risk factor for failed weaning from supplemental oxygen. Thus, major abdominal surgery patients need preemptive management to reduce postoperative hypoxemia. Despite inconsistent recommendations, postoperative continuous positive airway pressure can be an alternative to the facial mask [22]. In addition, preoperative desaturation during room air breathing also represented a significant risk for failed weaning. Preoperative hypoxemia is usually derived from preexisting respiratory impairments and is associated with postoperative respiratory complications [23]. Therefore, premature cessation of oxygen supplementation must be avoided in these patients if vigilant monitoring is not available.

From the perspective of anesthesia and analgesia, several studies reported that total intravenous anesthesia could facilitate comprehensive recovery after surgery compared to inhaled anesthesia despite low-certainty evidence [24, 25]. The advantage of total intravenous anesthesia for enhanced recovery might have contributed to successful weaning in this study. While overdosing on opioid administration induce respiratory depression, insufficient analgesia can cause tachypnea and disturb regular breathing, which can inhibit the recovery of adequate respiration and systemic oxygenation [26]. In other study on postoperative hypoxemia, Russell et al. [13] reported that old age, morbid obesity, higher ASA classification, and intraoperative fluid volume greater than 1,500 mL correlated with desaturation immediately postoperatively. Another study has identified longer duration of surgery, recent smoking history, and pre-existing comorbidities as significant predictors of postoperative hypoxemia [15]. Risk factors identified in these studies are unfavorable conditions for achieving and maintaining adequate spontaneous breathing and oxygenation postoperatively. We thought that successful weaning from supplemental oxygen therapy in our patients was inhibited through a similar mechanism to them.

The present study had several limitations. First, this was a retrospective cohort study based on the experience of a single center. Therefore, uncontrolled factors or biases may have influenced the results. Various clinical situations or methods of anesthesia and analgesia for each case that we could not consider in this study might have affected the weaning failure of supplemental oxygen. Second, the definition of hypoxemia varies among studies, but 90% SpO_2_ is generally used. However, we set 92% SpO_2_ as our threshold to determine postoperative hypoxemia and resume supplemental oxygen therapy at the PACU in our institute, considering the safety of patients during recovery from general anesthesia. Therefore, the result might have differed if the definition of postoperative hypoxemia was changed. Third, we did not assess hemodynamic variables such as blood pressure and heart rate during the PACU stay. Despite hemodynamics being managed to maintain within 20% of baseline values in most cases, hemodynamic aberrations during postanesthetic recovery might interrupt sufficient systemic oxygenation [27]. Therefore, the incidence and risk factors for failed weaning from supplemental oxygen therapy may be different after including hemodynamic variabilities during the PACU stay.

## Conclusions

In conclusion, an analysis of more than 12,000 general anesthetics revealed an overall risk of failed weaning from supplemental oxygen therapy of 1:14. The identified risk factors provide evidence to determine weaning from supplemental oxygen therapy at the PACU in patients who underwent general anesthesia.

## Data Availability

The datasets used and/or analyzed during the current study are available from the corresponding author on reasonable request.

## Abbreviations

ASA: American Society of Anesthesiologists
CI: Confidence interval
COPD: Chronic obstructive respiratory disease
NRS: Numeric rating scale
OR: Odds ratio
PACU: Postanesthesia care unit
SMD: Standard mean difference
SpO_2_: Peripheral oxygen saturation
